# High Glucose-Induced Oxidative Stress Increases the Copy Number of Mitochondrial DNA in Human Mesangial Cells

**DOI:** 10.1155/2013/754946

**Published:** 2013-07-30

**Authors:** Ghada Al-Kafaji, Jamal Golbahar

**Affiliations:** Diagnostic Services Unit, Al-Jawhara Centre for Molecular Medicine, College of Medicine and Medical Sciences, Arabian Gulf University, P.O. Box 26671, Manama, Bahrain

## Abstract

Oxidative damage to mitochondrial DNA (mtDNA) has been linked to the pathogenicity of diabetic nephropathy. We tested the hypothesis that mtDNA copy number may be increased in human mesangial cells in response to high glucose-induced reactive oxygen species (ROS) to compensate for damaged mtDNA. The effect of manganese superoxide dismutase mimetic (MnTBAP) on glucose-induced mtDNA copy number was also examined. The copy number of mtDNA was determined by real-time PCR in human mesangial cells cultured in 5 mM glucose, 25 mM glucose, and mannitol (osmotic control), as well as in cells cultured in 25 mM glucose in the presence and absence of 200 **μ**M MnTBAP. Intracellular ROS was assessed by confocal microscopy and flow cytometry in human mesangial cells. The copy number of mtDNA was significantly increased when human mesangial cells were incubated with 25 mM glucose compared to 5 mM glucose and mannitol. In addition, 25 mM glucose rapidly generated ROS in the cells, which was not detected in 5 mM glucose. Furthermore, mtDNA copy number was significantly decreased and maintained to normal following treatment of cells with 25 mM glucose and MnTBAP compared to 25 mM glucose alone. Inclusion of MnTBAP during 25 mM glucose incubation inhibited mitochondrial superoxide in human mesangial cells. Increased mtDNA copy number in human mesangial cells by high glucose could contribute to increased mitochondrial superoxide, and prevention of mtDNA copy number could have potential in retarding the development of diabetic nephropathy.

## 1. Introduction

Hyperglycemia is the most important factor causing the development of progressive diabetic nephropathy [1, 2]. In recent years, the mitochondrial dysfunctions and mitochondrial DNA (mtDNA) defects have been reported to be associated with the pathogenesis of diabetes and its complications. This diabetes-induced mtDNA defect has been highlighted in numerous studies by alteration of the mtDNA copy number [3–5]. Mitochondria are not only the main site of energy production, but also the major source of reactive oxygen species (ROS) generated as byproducts of oxygen metabolism. The human mitochondrial genome is a 16.6 kb circular double-stranded DNA with 1000–10,000 copies per cell [6], coding for proteins essential for cellular respiration and normal mitochondrial function.

Overproduction of mitochondrial superoxide during hyperglycemia has been postulated as the primary initiating mechanism that activates pathways of diabetes vascular tissue damage, leading to cellular redox imbalance and oxidative stress [7–9]. A number of *in vitro* and *in vivo* studies suggest that oxidative stress is increased in diabetic nephropathy [10–12]. In addition, overproduction of ROS by high glucose compromises the antioxidant defense mechanisms in diabetic nephropathy such as reduced levels of mitochondrial-specific manganese superoxide dismutase (MnSOD) and further aggravates oxidative stress [7, 8, 13]. Oxidative stress including ROS may damage mtDNA and impair electron transport chain, leading to more ROS production [14, 15]. It is suggested that mtDNA, owing to its close proximity to the electron transport chain, lack of histone protection, and less DNA repair capacity, is a primary target to ROS attack [15, 16].

The copy number of mtDNA that reflects the abundance of mitochondria in a cell may change under different energy demands and different physiological or environmental conditions [17]. It was observed that an increase in the mtDNA copy number per cell is associated with elevated oxidative stress in the human tissues of aged individuals [18, 19]. A positive correlation between elevated oxidative stress and mtDNA copy number was found in the blood of  hemodialysis patients [20]. The mtDNA copy number is also increased with increasing biomarkers of oxidative stress in human leukocytes [21].

In this study, we tested the hypothesis that high glucose-induced oxidative stress may increase the copy number of mtDNA in human mesangial cells and examined the effect of MnSOD mimetic on high glucose-induced mtDNA copy number.

## 2. Methods

### 2.1. Human Mesangial Cells Culture and Treatment

Human mesangial cells (ScienCell Research Laboratories) from the third to sixth passage were maintained under conditions previously described [22, 23]. For experimental studies, cells were grown to confluence, growth arrested in reduced serum (0.5% FBS) for 24 h, and were split into different experimental groups.

For treatment with different concentrations of glucose [22, 23], the cells were incubated in DMEM media (Sigma Aldrich) containing either 5 mM glucose (normal glucose) or 25 mM glucose (high glucose) for 24 h at 37°C. Osmotic controls included the cells incubated in identical experimental conditions with 5 mM glucose + 20 mM mannitol instead of 25 mM glucose.

For direct inhibition of mitochondrial superoxide radicals, the cells were incubated for 24 h with 25 mM glucose in the presence or absence of 200 *μ*M MnTBAP (Mn(III)tetrakis(4-benzoic acid)) porphyrin chloride; a cell permeable mimetic of manganese superoxide dismutase (MnSOD) from Biomol, Plymouth Meeting, PA [24]. Control cells were allowed to grow in 5 mM glucose. At the end of each experimental period, the cells were washed with Hanks' balanced salt solution (Gibco), harvested by trypsinization in 1 ml PBS and used for the preparation of genomic DNA.

### 2.2. Genomic DNA Preparation

Genomic DNA was extracted by QIAamp DNA Mini Kit (Qiagen) according to the manufacturer's protocol. In brief, 200 *μ*L lysis buffer was added to cell pellets, mixed, and incubated at 56°C for 10 min. Following centrifugation at 20,000 g for 1 min, absolute ethanol (200 *μ*L) was added and the lysate was centrifuged at 6000 g for 1 min, and washing procedures were carried out using washing buffers supplied with the kit. Genomic DNA was eluted by addition of elution buffer (200 *μ*L) and stored at −20°C. All genomic DNA samples were sheared prior to use.

### 2.3. Primers and Polymerase Chain Reaction (PCR)

Primers for NADH dehydrogenase subunit 2 (ND2) and cytochrome b (CYTB) genes were used as target sequences for determination of mitochondrial DNA. For the analysis of nuclear DNA, human beta-2-microglobulin (B2M) was selected as an internal reference gene. The primer sequences used for amplification of mitochondrial and nuclear DNA are listed in Table 1. The PCR reactions were performed in a total volume of 25 *μ*L reaction mixture, which contained DNA (1 *μ*g), forward and reverse primers (50 ng/*μ*L each), *Taq* DNA polymerase (1.25 U; Promega), MgCl_2_ (1.25 mM), 1X PCR reaction buffer, and NTPs (2.5 mM). Amplification reactions were carried out for 30 cycles using an automated thermal cycle (Perkin-Elmer 2400) under the cycling parameters of denaturation at 95°C for 5 min, annealing phase at 60°C for 1.5 min, and extension at 70°C for 7 min.

### 2.4. Cloning of PCR Products and Standard Preparation

The most convenient way to create a DNA standard is to clone a PCR product into a standard vector. For mtDNA and nDNA quantification by real-time PCR analysis, PCR products of ND2, CYTB, and B2M were purified using the GFX PCR DNA and gel band purification kit (GE Healthcare) and were cloned into the pGEM-T Easy Vector Systems (Promega) according to the manufacturer's instructions. The vectors were transformed into competent *E. coli* XL1 Blue cells and plasmid minipreps were prepared from selected positive clones using the Wizard plus SV Miniprep DNA Purification System (Promega). The clones were verified by sequencing (Geneservice), PCR amplification using gene-specific primers, and restriction enzyme digestion using *ApaI* and *PstI* (12 U/*μ*L each). For the generation of calibration curves, recombinant plasmids of  ND2, CYTB, and B2M were linearized by single digestion using *ApaI* restriction enzyme (12 U/*μ*L) and standard dilutions were prepared as previously described [22, 23], in which each gene is present in 10^7^ down to 10^3^ copies per *μ*L and was used as calibration curves in the real-time PCR runs.

### 2.5. Real-Time PCR

The copy number of mtDNA per nuclear genome was assessed by real-time PCR using the LightCycler System and the FastStart DNA Master SYBR Green 1 Kit (Roche Molecular Biochemicals). Total genomic DNA (10 ng) was mixed with SYBR Green 1 probe, forward and reverse primers (50 ng/*μ*L), and nuclease free water to the final volume of 10 *μ*L. The reactions were performed in 40 cycles of denaturation at 95°C for 10 min, amplification at 95°C for 10 sec, melting at 60°C for 10 sec, and cooling for 10 sec. The resulting data were analyzed using the Roche Molecular Biochemicals LightCycler software. The copy numbers of mtDNA and nDNA were calculated using the calibration curves that were constructed from dilution series of recombinant plasmids. The concentrations of mtDNA and nDNA were converted to copy numbers and calibration curves were plotted which are straight line  of  logarithm copy number (*x*-axis) and threshold cycle (*C*_*t*_) of the reaction (*y*-axis). Each measurement was carried out at least 3 times and normalized in each experiment against serial dilutions of a control DNA sample.

### 2.6. ROS Assay

Intracellular ROS production was detected in human mesangial cells using the redox-sensitive fluoroprobe carboxy-2′,7′-dichlorodihydrofluorescein diacetate (carboxy-H_2_DCFDA; Invitrogen) as previously described [22]. DCFDA is converted by intracellular esterases to 2′,7′-dichlorodihydrofluorescein, which, in turn, is oxidized by H_2_O_2_ to the highly fluorescent 2′,7′-dichlorodihydrofluorescein (DCF). Synchronized quiescent cells grown on glass coverslips were incubated with DMEM media (Sigma Aldrich) containing either 5 mM glucose or 25 mM glucose for 24 h at 37°C. The cells were washed with PBS and incubated in the dark for 30 min with 25 *μ*M carboxy-H_2_-DCFDA dissolved in the culture media. For the positive control, cells incubated with 5 mM glucose were treated with 100 *μ*M tetra-butyl hydroperoxide (TBHP) (Invitrogen) for 1 h, washed and loaded with carboxy-H_2_-DCFDA. All cells were examined using a laser scanning confocal microscope (LSM 510; Zeiss, Oberkochen, Germany). H2DCFDA was detected on excitation of 488 nm and emission of 525 nm.

In addition, a FACScan flow cytometer (Becton Dickinson, Bedford, MA, USA) equipped with a 488 nm argon laser was used for the flow-cytometric analysis to monitor intracellular ROS generation in human mesangial cells [22]. In brief, cells were incubated with 5 mM glucose or 25 mM glucose for 24 h at 37°C, washed and loaded with carboxy-H_2_-DCFDA (25 *μ*M, 30 min) as above. Positive control cells were incubated with 5 mM glucose in the presence of TBHP (100 *μ*M) for 1 h, washed and loaded with carboxy-H_2_-DCFDA. After incubation, cells were resuspended in PBS and used for flow cytometry analysis using an excitation wavelength of 488 nm and an emission wavelength of 525 nm.

### 2.7. MitoSOX Red Analysis

The production of mitochondrial superoxide in human mesangial cells, was detected using MitoSOX red fluorogenic dye (Invitrogen). MitoSOX red permeates live cells, where it selectively targets mitochondria and is readily oxidized by superoxide. Synchronized quiescent cells grown on glass coverslips were incubated for 24 h at 37°C with 25 mM glucose in the presence and absence of 200 *μ*M MnTBAP. Control cells were maintained in 5 mM glucose. At the end of the incubation, MitoSOX (5 *μ*M) was added to the cells and incubated further for 20 min at 37°C. The cells were washed with PBS and used for confocal microscopy. MitoSOX red was excited by laser at 514 nm and mean fluorescence intensity per square millimeter cell area was calculated using Zeiss software.

### 2.8. Statistical Analysis

Student's *t*-test was used to compare the copy number of ND2 and CYTB per B2M in different conditions. Results were expressed as mean ± SD. All reported *P* values are two tailed and a *P* < 0.05 was considered to be statistically significant. The statistical analyses were performed using SPSS software, version 19.

## 3. Results

### 3.1. Verification of mtDNA and nDNA Inserts into pGEMT

The lengths of mtDNA and nDNA inserts were verified by PCR amplification and restriction enzyme digestion. The agarose gel electrophoresis result of PCR amplifications showed specific PCR products of the expected lengths of 89-bp, 165-bp, and 182-bp that correspond to ND2, CYTB, and B2M, respectively, confirming the size of each insert (data not shown). In addition, the restriction enzyme analysis showed fragments of the same lengths of ND2, CYTB, and B2M inserts (data not shown).

### 3.2. Efficiency of Quantitative Real-Time PCR

For obtaining efficient amplification results, recombinant plasmids of ND2, CYTB, and B2M were linearized and calibration curves were generated from duplicate measurements of 5 independent serial dilutions of each individual plasmid. The real-time PCR efficiency of each calibration curve was determined by plotting the *C*_*t*_ value against the log template amount. From the slope (*S*), the efficiency was calculated using the following formula:

(1) $$\begin{array}{c} \text{efficiency}\left(\%\right)=\left(10^{\left(-1/S\right)}-1\right)\times 100. \end{array}$$

The corresponding amplification efficiencies, 94–100%, were in the optimum range with a standard error mean of less than 0.05 (Table 2).

### 3.3. Effect of Glucose on mtDNA Copy Number

**Figure 1:**
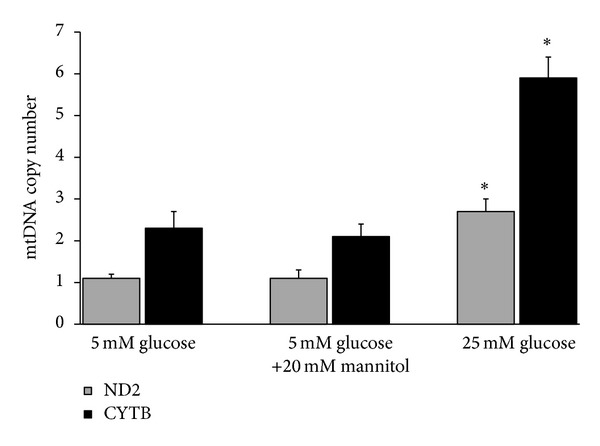
Effect of high glucose on mtDNA copy number. Synchronized quiescent mesangial cells were incubated with 5 mM glucose, 25 mM glucose, and 5 mM glucose plus 20 mM mannitol for 24 h. The copy numbers of ND2 and CYTB per B2M were determined by real-time PCR. Values represent mean ± SD of three independent experiments. *P < 0.05 compared to 5 mM glucose and to 5 mM glucose plus 20 mM mannitol.

The effect of high glucose on mtDNA copy number in human mesangial cells is shown in Figure 1. The copy numbers of ND2 and CYTB per B2M were significantly increased by 2.5- and 2.6-folds, respectively, when the cells were incubated with 25 mM glucose compared to 5 mM glucose (*P* < 0.05). Incubation of cells in 5 mM glucose + 20 mM mannitol (instead of 25 mM glucose) did not have any effect on mtDNA copy number, suggesting that the effects observed under high glucose conditions were not induced by increased osmolarity. The mean ND2 copy number per copy of B2M gene was 2.7 ± 0.3 in 25 mM glucose compared to that of 5 mM glucose (1.1 ± 0.1) and 5 mM glucose + 20 mM mannito (1.1 ± 0.2). Similarly, the mean CYTB copy number per copy of B2M gene was 5.9 ± 0.5 in 25 mM glucose compared to that of 5 mM glucose (2.3 ± 0.4) and 5 mM glucose + 20 mM mannitol (2.1 ± 0.3).

### 3.4. Accumulation of ROS in High Glucose-Treated Human Mesangial Cells

**Figure 2:**
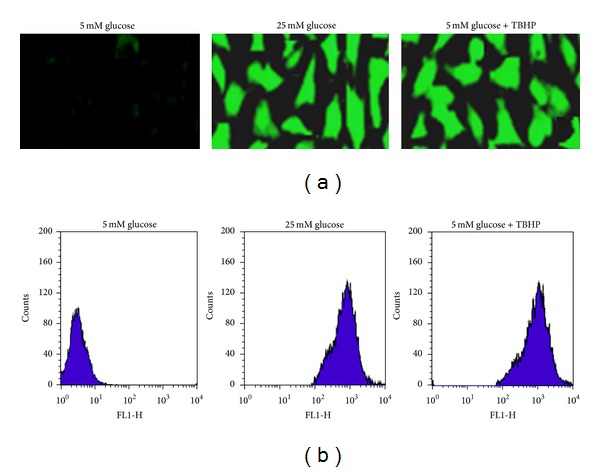
High glucose-induced ROS generation. Synchronized quiescent mesangial cells grown on glass coverslips were incubated for 24 h with 5 mM glucose or 25 mM and loaded with carboxy-H_2_-DCFDA (25 *μ*M, 30 min). Positive control cells were incubated with 5 mM glucose, and treated with TBHP (100 *μ*M, 1 h), washed and loaded with carboxy-H_2_-DCFDA (25 *μ*M, 30 min). Intracellular ROS was detected by confocal microscopy and flow cytometry. (a) Representative confocal images of ROS generation in human mesangial cells. The fluorescent intensity of DCF corresponds to the extent of ROS accumulation. (b) Representative histograms of flow cytometry demonstrating the fluorescent intensity of DCF in human mesangial cells (*n* = 3).

The accumulation of intracellular ROS by high glucose in human mesangial cells was assessed by confocal microscopy and flow cytometry using the redox-sensitive fluoroprobe carboxy-2′,7′-dichlorodihydrofluorescein diacetate (carboxy-H_2_DCFDA). As shown in Figures 2(a) and 2(b), production of ROS was observed following exposure of the cells to 25 mM glucose as detected by higher DCF-fluorescent intensity compared to 5 mM glucose. Furthermore, a strong DCF-fluorescent signal was detected in the positive control cells that were maintained in 5 mM glucose and treated with tetra-butyl hydroperoxide (TBHP) compared to cells maintained in 5 mM glucose alone.

### 3.5. Effect of MnTBAP on mtDNA Copy Number

**Figure 3:**
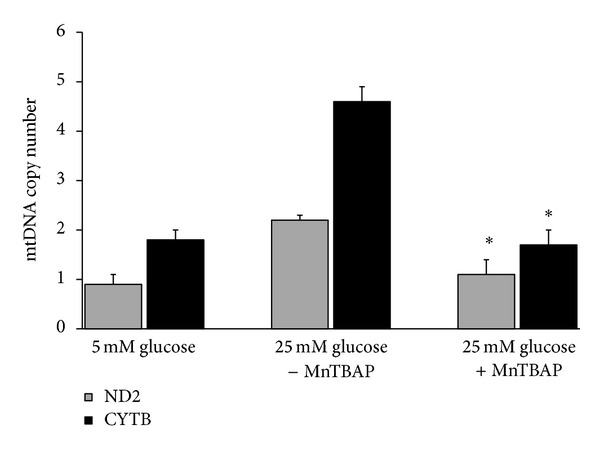
Effect of MnTBAP on high glucose-induced mtDNA copy number. Synchronized quiescent mesangial cells were incubated with 25 mM glucose in the presence or absence of MnTBAP (200 *μ*M) or 5 mM glucose for 24 h. The copy numbers of ND2 and CYTB per B2M were determined by real-time PCR. Values represent mean ± SD of three independent experiments. *P < 0.05 compared to 25 mM glucose without MnTBAP.

The effect of direct inhibition of mitochondrial superoxide by MnTBAP on mtDNA copy number induced by high glucose in human mesangial cells is shown in Figure 3.

Addition of MnTBAP during high glucose incubation prevented the increase in mtDNA copy number, as the mean ND2 copy number per copy of B2M gene was 1.1 ± 0.3 and was significantly decreased by 2.0-fold when the cells were maintained in 25 mM glucose plus MnTBAP compared to that of 25 mM glucose without MnTBAP (2.2 ± 0.1) (*P* < 0.05). In a similar manner, the mean CYTB copy number per B2M copy number gene (1.7 ± 0.3) was significantly decreased by 2.7-fold when the cells were incubated with 25 mM glucose in the presence of MnTBAP compared to cells incubated with 25 mM glucose in the absence of MnTBAP (4.6 ± 0.3) (*P* < 0.05).

### 3.6. Inhibition of High Glucose-Induced Mitochondrial Superoxide in Human Mesangial Cells

**Figure 4:**
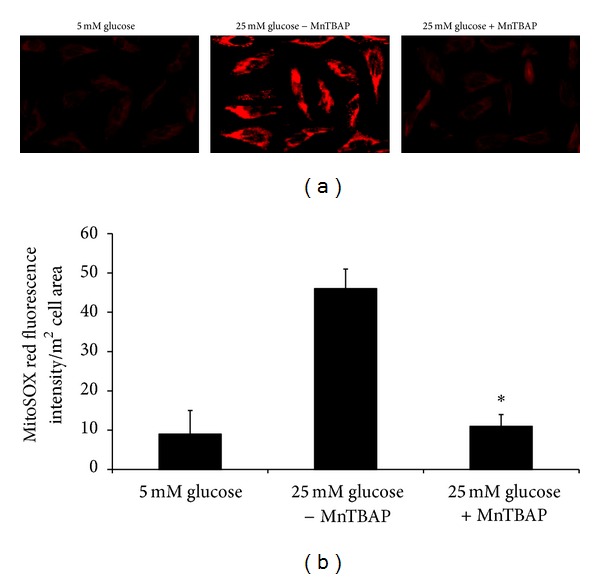
Inhibition of mitochondrial superoxide by MnTBAP in human mesangial cells. Synchronized quiescent mesangial cells grown on glass coverslips were incubated for 24 h with 25 mM glucose with and without MnTBAP (200 *μ*M) or 5 mM glucose, loaded with MitoSOX (5 *μ*M, 20 min), and examined by confocal microscopy. (a) Representative confocal images of human mesangial cells showing decrease in mitochondrial MitoSOX fluorescence following incubation of the cells with 25 mM glucose and MnTBAP. (b) Quantitative analysis of the MitoSOX fluorescence intensity per square millimeter cell area in human mesangial cells. Values represent mean ± SD of three independent experiments. *P < 0.05 compared to 25 mM glucose without MnTBAP.

The direct inhibition of superoxide produced by mitochondria under high glucose conditions in human mesangial cells is shown in Figure 4.

Confocal microscopic imaging demonstrated markedly reduced mitochondrial fluorescence intensity of MitoSOX red when human mesangial cells were cultured in 25 mM glucose and treated with MnTBAP compared to 25 mM without MnTBAP and in control cells cultured in 5 mM glucose (Figure 4(a)). A quantitative analysis of the mean intensity indicated a significant decrease in the fluorescence of MitoSOX red at 25 mM glucose and MnTBAP treatment compared to 25 mM glucose without MnTBAP treatment (*P* < 0.05) (Figure 4(b)).

## 4. Discussion

Each human cell contains several hundreds to more than a thousand mitochondria. The copy number of mtDNA varies in different cell types, and when physiological or environmental conditions are changed, mtDNA copy number can be modulated [17]. Results from this *in vitro* study showed that the copy number of mtDNA is increased in human mesangial following 24 h incubation in high glucose, and this increase may be due to oxidative stress induced by high glucose. In support of this suggestion, we observed that inclusion of MnSOD mimetic (MnTBAP) during high glucose incubation prevented the increase in mtDNA copy number.

High glucose-induced mitochondrial ROS production has been previously reported in human renal mesangial cells [9–11, 22]. In this study, the accumulation of ROS in human mesangial cells cultured under high glucose conditions was confirmed by increased intensity of DCF-sensitive fluorescence in the cells. Furthermore, the production of mitochondrial ROS by high glucose was confirmed by an increase in fluorescence intensity of the specific mitochondrial MitoSOX red probe, which was inhibited by the addition of MnTBAP during high glucose incubation.

These observations consolidate the results, in which high glucose increases intracellular ROS in human mesangial cells, and support the hypothesis that increased mtDNA copy number of  human mesangial cells is caused by oxidative stress resulting from high glucose.

Overproduction of ROS by the mitochondrial respiratory chain may damage mtDNA that is more susceptible to oxidative stress than nuclear DNA [14, 15]. As a result, the mitochondrial function is compromised, and consequently, ROS production is further increased causing more oxidative damage to mitochondria and mtDNA [15, 16].

It has been shown that mtDNA harboring deleterious mutations are preferentially clonally amplified as a compensatory response to energy deficiency by making more mitochondria and mtDNA [25]. Lee et al. [26] reported an increase in mtDNA content following treatment of human lung fibroblasts with H_2_O_2_, which is consistent with the proliferation of mitochondria. An increase in the mitochondrial mass was also observed in skin fibroblasts of myoclonic epilepsy patients compared with those of normal skin fibroblasts [27]. The increase in mitochondrial mass and mtDNA content may be an early molecular event of human cells in response to endogenous or exogenous oxidative stress [26].

The observation in this study that mtDNA copy number is increased in human mesangial cells by high glucose-induced oxidative stress may partially substantiate the evidence of increased mtDNA copy number reported in the blood of diabetic nephropathy patients [5]. Hyperglycemia-induced overproduction of mitochondrial ROS is considered as the central molecular mechanism that activates various biochemical pathways postulated to be implicated in the development of vascular complications in diabetes that include diabetic nephropathy, and increased oxidative stress is believed to be an important contributor [7–9].

It has been shown that hyperglycemia leads to an early inactivation of MnSOD, which in turn increases mitochondrial oxidant production and precedes kidney dysfunction in rat model of diabetic nephropathy [28]. Furthermore, normalizing the levels of mitochondrial ROS with MnSOD prevents the activation of hyperglycemic vascular damaging pathways in diabetes [8], and overexpression of MnSOD suppresses high glucose-induced collagen accumulation in cultured mesangial cells [29].

Hyperglycemia is reported to induce mitochondrial dysfunction, mtDNA oxidative damage, and apoptosis in experimental and animal models of diabetic retinopathy [24, 30, 31]. In animal models of diabetic complications, Kakimoto et al. [32] showed a major role of oxidatively damaged mtDNA in the pathogenesis of diabetic nephropathy.

Increased numbers of mtDNA copies in human mesangial cells by high glucose observed in this study may be a compensatory response mechanism to mtDNA damage and a decline in the electron transport system caused by oxidative stress. At the same time, superoxide and other ROS would be generated from the increased mitochondria in these cells. Consequently, it can cause more oxidative damage to mtDNA and mitochondria with further production of ROS [14]. The beneficial effect of MnSOD mimetic in preventing high glucose-induced mtDNA copy number in human mesangial cells supports a critical role of MnSOD in protecting mtDNA from oxidative injury in diabetic nephropathy. MnSOD is mainly responsible for metabolism of superoxide produced in mitochondria by respiratory chain activity during aerobic metabolism of glucose and other substrates. It is the primary antioxidant enzyme that protects cells from oxidative stress by catalyzing dismutation of superoxide to hydrogen peroxide and oxygen in the mitochondria of eukaryotic cells.

One of the limitations in this study is the lack of time-course experiments examining the alteration in mtDNA copy number in high glucose cultured-human mesangial cells. In addition, translation of the study findings in the human context is difficult, given that the experimental conditions, mainly exposure to glucose as high as 25 mM, are unusual finding in patients with diabetes.

In conclusion, this is the first study to show an increase in mtDNA copy number by high glucose in human mesangial cells that might be a biological adaptation in response to oxidative stress. Protection of mtDNA copy from glucose-induced mitochondrial ROS by MnSOD could have potential in retarding the development of diabetic nephropathy. Further studies are required to investigate the role of mtDNA copy number in the pathogenesis of diabetic nephropathy.

## Figures and Tables

**Table 1:** Primers used for the amplification of mtDNA (ND2, CYTB) and nDNA (B2M).

Gene	Sequence forward primer 5′ to 3′	Sequence reverse primer 5′ to 3′	Length (bp)
NADH dehydrogenase subunit 2 (ND2)	CACAGAAGCTGCCATCAAGTA	CCGGAGAGTATATTGTTGAAGAG	89
Cytochrome b (CYTB)	TCATCGACCTCCCCACCCCATC	CGTCTCGAGTGATGTGGGCGATT	165
Beta-2-microglobulin (B2M)	TGGCCATACTACCCTGAATGAGTCC	ATGTATTGTGCAATGCTGCTGCTCG	182

**Table 2:** Real-time PCR efficiency of mtDNA and nDNA standards.

Gene	Slope	Efficiency (%)	Error
ND2	−3.374	94	0.041
CYTB	−3.31	99	0.020
B2M	−3.30	100	0.010
